# Clinical and Functional Outcomes of Knee Arthrodesis by Charnley’s External Fixator

**DOI:** 10.7759/cureus.72233

**Published:** 2024-10-23

**Authors:** Manish R Shah, Aumkar H Pandya, Richenkumar R Shah, Isha M Shah

**Affiliations:** 1 Orthopaedics, Sumandeep Vidyapeeth Deemed to be University, Vadodara, IND; 2 Orthopaedics, Smt. B. K. Shah Medical Institute and Research Centre, Sumandeep Vidyapeeth Deemed to be University, Vadodara, IND; 3 Orthopaedics, Gujarat Medical Education and Research Society (GMERS) Medical College, Gotri, Vadodara, IND

**Keywords:** charnley’s fixator, compression arthrodesis, knee arthrodesis, knee fusion, knee joint fusion

## Abstract

Background

Knee arthrodesis was originally developed to manage severe joint infections such as tuberculosis before the advent of antibiotics and joint replacement techniques. The procedure aims to eliminate pain and infection by stabilizing the knee joint through bone fusion. Knee arthrodesis remains essential for cases where total knee arthroplasty is not feasible, such as in patients with severe bone loss, chronic infections, or failed knee replacements. Many studies have demonstrated fusion by external fixators because infection is the most common indication of this procedure. We studied knee arthrodesis using Charnley’s compression clamps which are easy to apply and economical.

Methodology

A prospective observational study was conducted on 15 patients for 18 months. Patients with an infection in the knee joint (septic or tuberculous), failed total joint arthroplasty, failed fracture fixation around the knee joint, and neuropathic joints were included in the study. Patients were evaluated using the pre and postoperative Knee Society Score and Knee Society Score (Functional).

Results

In the study, 80% of the patients were males. Septic arthritis and tuberculosis were common indications for the surgery. More than 70% of the patients were previously operated on the same knee joint. The duration of the fixator ranged from 32 weeks to 39 weeks (7.47 months to 9.1 months) with an average of 35.73 weeks (8.34 months). The final follow-up period ranged from 9.5 to 18 months with an average of 14.1 months. The knee score ranged preoperatively from 5 to 22 (average = 16.2) and improved postoperatively ranging from 50-56 (average = 54). The functional score ranged preoperatively from 40 to 50 (average = 43.60) and improved postoperatively ranging from 80 to 100 (average = 89.66).

Conclusions

The fusion by Charnley’s external clamps is easy, cost-effective, and comfortable for the patients. The complications are relatively fewer and the success rate of the fusion is higher with this treatment method.

## Introduction

Knee arthrodesis, or knee fusion, is a known procedure in orthopedic surgery since the beginning of the 20th century. It was originally developed to manage severe joint infections such as tuberculosis before the advent of antibiotics and joint replacement techniques. The procedure aims to eliminate pain and disease (infection) by stabilizing the knee joint through bone fusion. The only option in some cases can be amputation [1]. However, with the development of total knee arthroplasty (TKA) in 1970, the use of knee arthrodesis declined as TKA provided better joint function while preserving mobility. Despite this, knee arthrodesis remains essential for cases where TKA is not feasible, such as in patients with severe bone loss, chronic infections, or failed knee replacements [2].

Today, it is considered a specialized surgical option, reserved for complex cases that cannot be managed by routine treatment options. Over time, surgical techniques evolved, and by the mid-20th century, the introduction of internal fixation methods, such as nails, plates, and screws, enhanced the success of knee arthrodesis by improving joint stabilization during healing [3]. There is no consensus about the type of external fixator for knee joint arthrodesis. Unilateral, bi-planar, and circular fixators have been used for arthrodesis [4,5].

Many studies have demonstrated fusion by external fixators because infection is the most common indication of this procedure [4,6,7]. Fewer recent studies demonstrate arthrodesis only by compression using a unilateral external fixator. We studied knee arthrodesis by using Charnley’s compression clamps. The device is easy to apply and economical. This study explores the clinical functional outcomes of knee arthrodesis, focusing on pain relief, gait patterns, and overall functional capabilities. By examining these outcomes and associated complications, the study aims to provide insights that will assist orthopedic surgeons and healthcare professionals in making informed decisions, ultimately improving patient care in challenging knee conditions.

## Materials and methods

The prospective observational study was conducted among 15 patients admitted to the institute. Approval from the Ethical Committee of Sumandeep Vidyapeeth, Vadodara, Gujarat, India was obtained (approval number: SVIEC/ON/MEDI/RP 24/25). All patients operated on for knee joint arthrodesis by Charnley’s compression clamps were included after meeting the inclusion and exclusion criteria. Written and informed consent were obtained before the surgery. The study was conducted from April 2023 to September 2024.

All patients aged over 18 years who consented to participate in the study were included. All patients with an infection in the knee joint (septic or tuberculous), failed total joint arthroplasty, failed fracture fixation around the knee joint, and neuropathic joints were included in the study. All patients who refused to participate in the program, follow-up of fewer than six months, arthrodesis due to bone malignancy, bilateral knee involvement, and ipsilateral hip joint involvement were excluded from the study.

All previous treatment records were obtained. After preoperative X-rays, patients were counseled about the non-viability of other options that can preserve the knee joint movement. They were explained about the procedure considering no knee joint movements and shortening (to be compensated by shoe raise). All patients were given long knee braces and allowed one day (during the preoperative fitness period) to make the final decision of knee joint fusion. Written and informed consent (patients were informed of arthrodesis and expected problems in day-to-day living) was taken and recorded. After the final consent of the patients and fitness from an anesthesia point of view, they were operated on. All patients were operated by a single surgeon with experience of more than 25 years.

Operative technique

All standard aseptic and antiseptic precautions were taken. All patients were operated on in a supine position on a standard operation table. Sinus tracks were defined by methylene blue injection. A midline skin incision was made. The sinus opening was incorporated by an eye-shaped incision (if the sinus was not in the midline, an elliptical incision including the sinus opening was taken separately). Thorough debridement was done. A deep culture was taken in case of infection. Infected fluid and tissue were sent for bacterial and fungal cultures. Excised tissue was sent for histopathology examination. The specimen was sent for GeneXpert in suspected cases of tuberculosis.

**Figure 1:**
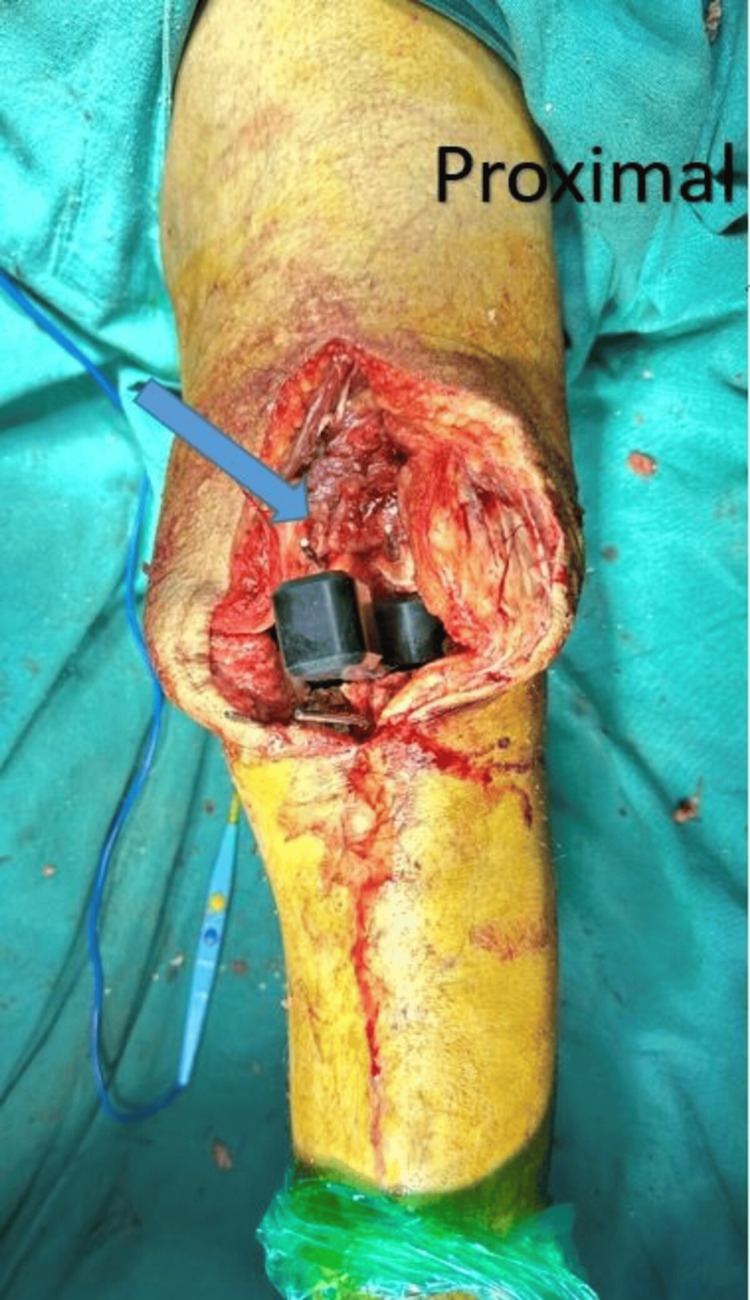
Intraoperative image. The arrow shows the extension block for balancing.

After a thorough wash with a pulse lavage system, a distal cutting jig for the femur and a proximal cutting jig for the tibia (instruments set from the TKA set) were used to cut the distal femur and proximal tibia. Extension balance was checked by the block used in TKA (Figure 1). Alignment was checked by alignment rods (similar to TKA). Dunham’s pins (4 mm or 4.5 mm as per the build of the patient) were inserted in the femur and tibia. Charnley’s compression clamps were attached to Dunham’s pins. Fusion was done in 5° valgus, neutral extension, and 3° external rotation. Subsequently, 5 mm compression was provided by the clamps on each side, and the fusion was checked in the image intensifier (IITV). The wound was closed in layers. Sterile dressing including pin-tracks was applied.

**Figure 2:**
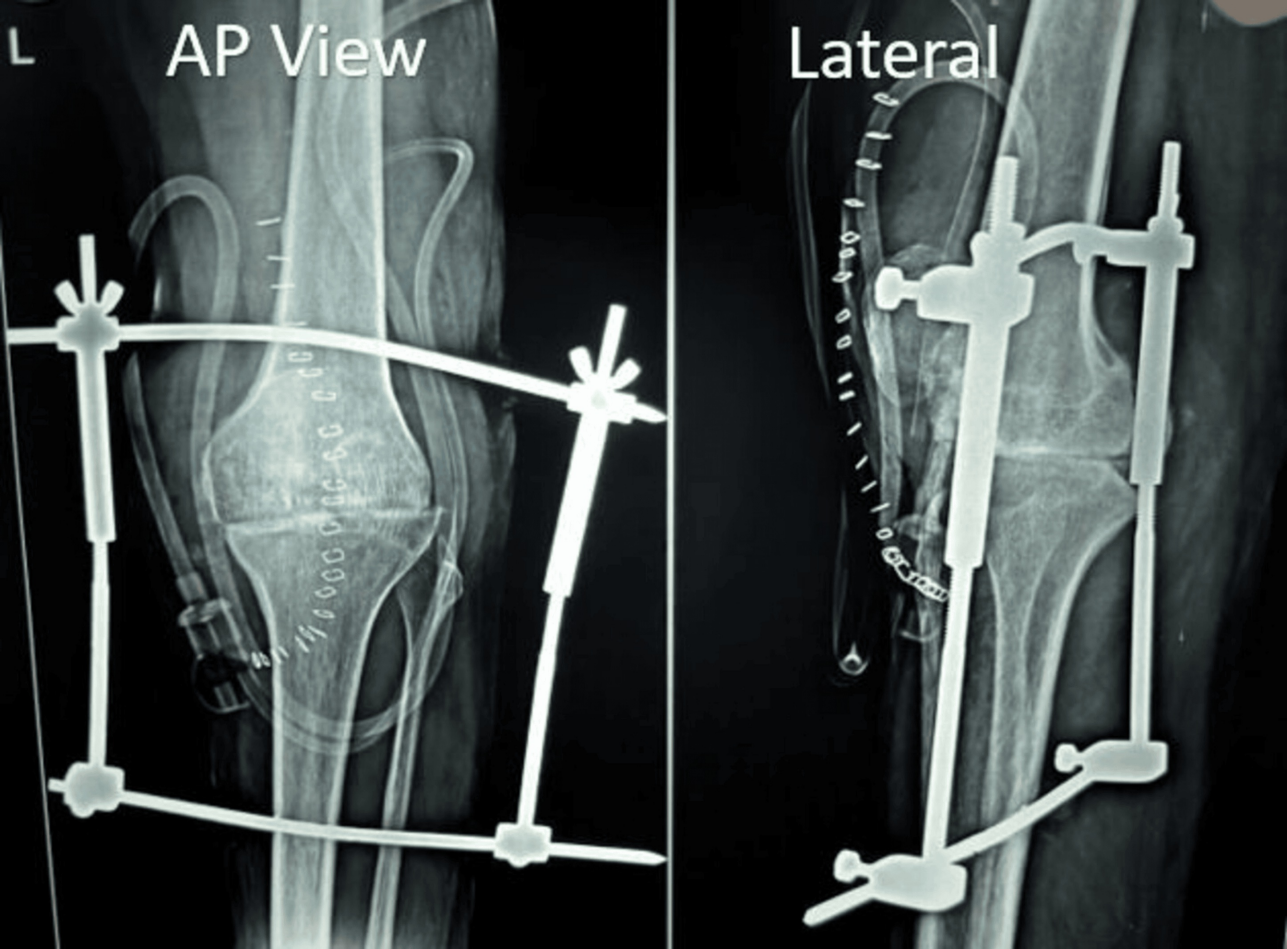
Immediate postoperative X-ray.

Postoperative X-rays were obtained on the same day (Figure 2).

**Figure 3:**
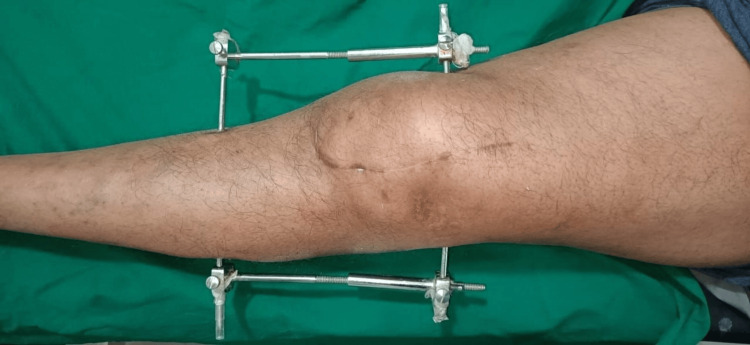
Clinical photograph after suture removal.

Intravenous (IV) antibiotics (piperacillin + tazobactam 4.5 g and amikacin 500 mg) were continued till the report of culture and sensitivity was available. Antibiotics were changed later as per the culture and sensitivity report. In culture-negative cases, oral antibiotics (cefixime 200 mg twice a day) were given until suture removal (the usual duration of antibiotics in culture-negative cases was a maximum of three weeks). Patients were taught static quadriceps exercises from the first postoperative day. All patients were allowed to touch weight bearing (approximately 10% body weight only) with a walker and shoe raise from the first postoperative day. The first dressing was done on the fifth postoperative day and the second dressing on the 10th postoperative day. The patient was discharged if the wound was clean on the 10th postoperative day. Suture removal was done at around three weeks (Figure 3). Patients were taught pin-track dressings (patients were given sterile gauze packs and taught to clean the pin tracks with hydrogen peroxide and apply povidone-iodine dressing) after suture removal and were called for monthly follow-up or in case of any signs of infection. Blood reports were repeated at the time of discharge, at the time of suture removal, and at each follow-up in infected cases. Usually, the antibiotics were continued until blood parameters were near normal and there were no clinical signs of infection. For non-tuberculous cases, it was usually 10-12 weeks. For tuberculous cases, the regimen was for one year as per the standard recommendation for osteoarticular tuberculosis. In proven tuberculosis cases, a standard antituberculous regime was given and the patients were monitored as per standard guidelines for tuberculosis treatment.

**Figure 4:**
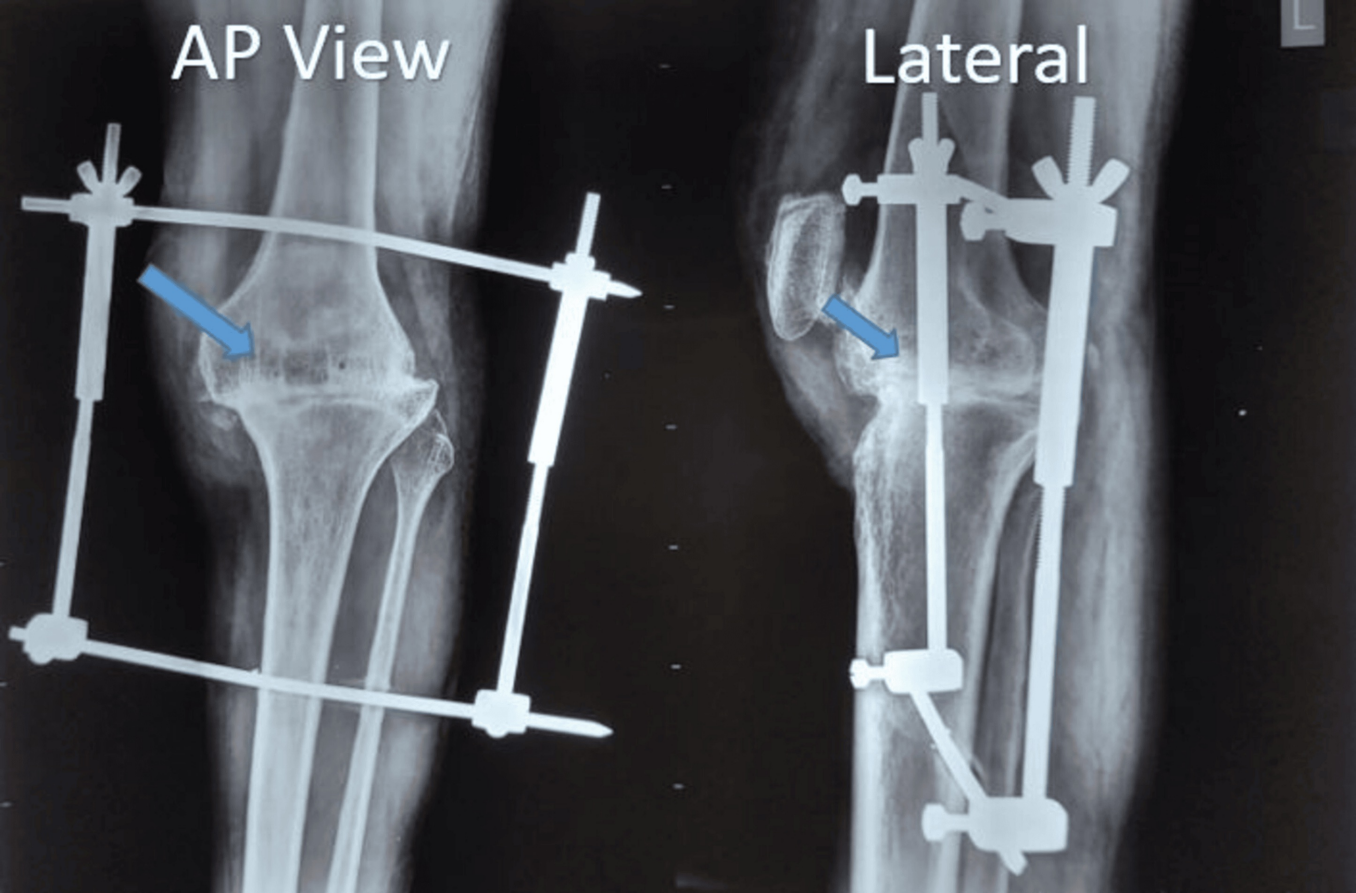
Radiological union. The arrows show the continuity of trabeculae.

The radiological union was considered when the trabecular pattern was found in continuity in the tibia and femur in two out of four views in anteroposterior and lateral views of the knee joint (Figure 4).

**Figure 5:**
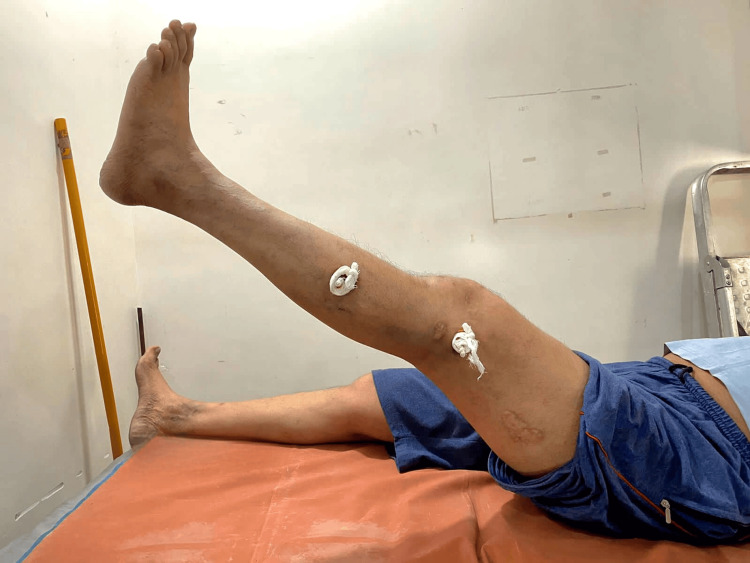
Patient performing the straight leg raise test after the removal of external clamps.

The external clamps were removed at this stage and the clinical union was checked. If the union had good clinical and radiological parameters, the full leg cast was applied for one month and the patient was allowed to fully weight bear with a walker. After one month, the plaster was removed. If clinical and radiological parameters confirmed solid union, the patient could perform an active straight leg raising test (Figure 5), and the patient could stand on an operated leg at least for 30 seconds without support, the pins were removed. The patient was allowed to walk with full weight bearing using a shoe raise as per requirement in individual cases. Patients were advised regular follow-ups at least twice in year. Patients were evaluated using the pre and post-operative Knee Society Score and Knee Society Score (Functional) using an online calculator (https://orthotoolkit.com/knee-society-score/).

All the data were entered in Microsoft Excel (Microsoft Corp., Redmond, WA, USA) and exported to SPSS Version 23 (IBM Corp., Armonk, NY, USA) for data analysis. All quantitative data were presented as mean and standard deviation (SD). The qualitative data were presented as numbers and percentages. The paired t-test was used to determine statistical significance in pre and postoperative knee scores and pre and postoperative knee society scores. P-values <0.05 were considered significant.

## Results

We found a majority (80%) of male patients in our study (12/15). The majority (80%) of them were in the age group of 51 to 70 years. The left knee joint was involved in 60% (9/15) of cases and the right knee was involved in 40% (6/15) of cases. Septic arthritis and tuberculosis were the common indications (33.33% each) for surgery (five each). Other indications were failed total knee replacement (TKR) (20%), rheumatoid arthritis (end-stage bone and joint destruction with instability and the joint preservation was likely to fail) (6.66%), and neuropathic joint (6.66%). Two (13.33%) patients had diabetes mellitus and one (6.66%) had hypertension. All demographic data are shown in Table 1.

**Table 1:** Patient demographics. DM = diabetes mellitus; HT = hypertension; TKR = total knee replacement

Number of patients (n = 15)		
Sex	Male	12 (80%)
	Female	3 (20%)
Age (years)	40–50	1 (6.66%)
	51–60	5 (33.33%)
	61–70	7 (46.66%)
	71–80	2 (13.33%)
Side	Right	6 (40%)
	Left	9 (60%)
Comorbidities	DM	2 (13.33%)
	HT	1 (6.66%)
Indication for arthrodesis	Septic knee	5 (33.33%)
	Tuberculous knee	5 (33.33%)
	Failed TKR	3 (20%)
	Rheumatoid arthritis	1 (6.66%)
	Neuropathic joint	1 (6.66%)

Overall, 11 out of 15 patients were previously operated on the same knee joint. Five (33.33%) patients had undergone one surgery, five (33.33%) patients had undergone two surgeries, and one (6.67%) patient had undergone three surgeries. Patients were treated in the form of joint aspiration, debridement, implant extraction, and spacer application before the decision of arthrodesis (Table 2).

**Table 2:** Number of surgeries before arthrodesis.

Surgery before arthrodesis	Number	%
0	4	26.67
1	5	33.33
2	5	33.33
3	1	6.67

The average duration of hospital stay was 12 days (range = 10 to 14 days). The duration of the fixator (day of surgery to pins removal) ranged from 32 to 39 weeks (7.47 months to 9.1 months) with an average of 35.73 weeks (8.34 months). The shortest duration of fixator removal was in septic arthritis cases and the longest was in rheumatoid arthritis cases (Table 3).

**Table 3:** Time for fixator removal and indications. TKR = total knee replacement

Indication for arthrodesis	Average time of fixator removal (weeks)
Septic arthritis	34.2
Tuberculous arthritis	35.6
Failed TKR	37.3
Rheumatoid arthritis	39
Neuropathic joint	36

The final follow-up period ranged from 9.5 to 18 months with an average of 14.1 months. The patients were evaluated at the final follow-ups using the Knee Society Score. It has two components, namely, a knee score and a functional score. The knee score ranged preoperatively from 5 to 22 (average = 16.2) and improved postoperatively from 50 to 56 (average = 54). The functional score ranged preoperatively from 40 to 50 (average = 43.60) and improved postoperatively from 80 to 100 (average = 89.66) (Tables 4, 5).

**Table 4:** Knee Society Score.

	Preoperative	Postoperative
Range	5–22	50–56
Average	16.2	54

**Table 5:** Knee Society Score (Functional).

	Preoperative	Postoperative
Range	40–50	80–100
Average	43.6	89.66

All patients had sound fusion as confirmed by clinical and radiological assessment. Two out of 15 patients had superficial tibial pin-track infections. Methicillin-resistant *Staphylococcus aureus* was the most common organism in septic arthritis cases. *Streptococcus*, *Escherichia coli*, and *Klebsiella* were the other organisms in such cases. Both pin-track infection cases were diabetic with above-normal blood sugar levels in the postoperative period. Infection was controlled by regular dressings and antibiotics. Both patients had sound arthrodesis at the final follow-up. The average shortening was 2.5 inches and was compensated by a shoe raise. We used Dunham’s pins, which are serrated in the middle for good bone purchase. Hence, pin loosening was not observed in any of our cases. All patients were satisfied with the treatment and none required any other surgical intervention on the same knee. All were able to walk without a walking aid.

We observed that the mean preoperative Knee Society Score was 16.20 ± 5.33 and the mean postoperative Knee Society Score was 54.00 ± 2.27 among enrolled patients. We applied the paired t-test to check the statistical pre and post-mean differences. The p-value of 0.0001 showed that this mean difference was statistically significant (Table 6).

**Table 6:** Statistical analysis of the Knee Society Score.

Knee Society Score	Mean	SD	P-value
Preoperative	16.20	5.33	0.001
Postoperative	54.00	2.27	

We observed that the mean preoperative Functional Knee Score was 46.60 ± 3.62 and the mean post-operative Functional Knee Score was 89.66 ± 7.89 among enrolled patients. We applied the paired t-test to check the statistical pre and postoperative mean difference. A p-value of 0.0001 indicated that this mean difference was statistically significant (Table 7).

**Table 7:** Statistical analysis of the Functional Knee Score.

Functional Knee Score	Mean	SD	P-value
Preoperative	43.60	3.62	0.001
Postoperative	89.66	7.89	

## Discussion

Knee arthrodesis is an old accepted procedure mainly indicated when all other options for the preservation of knee joint movements are not viable. The earliest documented cases of knee arthrodesis primarily focused on managing joint infections, with initial methods emphasizing the use of bone grafts and simple immobilization techniques. However, with the advancements in surgical practices and the introduction of internal fixation devices, the effectiveness of the procedure has improved significantly.

Figgie et al. [2] reported fusion in 20 out of 23 patients (27 knees) with rheumatoid arthritis. They used internal or external fixators. They showed the failure of arthrodesis in case of persistent sepsis and poor bone stock cases.

In the study by Brown et al. [3], antegrade intramedullary nail was a viable option for knee arthrodesis for chronically infected TKR. They showed successful fusion in 16 out of 17 (94%) patients. Overall, 59% of the patients in their study were able to ambulate with an assist device. However, 41% required a wheelchair. They showed complications in 47% of patients and 33% of their patients died within two years of the fusion procedure.

Eralp et al. showed arthrodesis by unilateral external fixator [4]. Infection of the joint was the common indication for arthrodesis in their study. They demonstrated better results with unilateral external fixators compared with circular external fixators in 11 patients. Except for two patients, the average shortening in their study was 1.4 cm. They demonstrated pin-track infections in five patients, which were treated with antibiotics and wound care. The total fixator time in their study ranged from 5 to 12 months (average = 8 months). They concluded that a monolateral external fixator is better in terms of patient comfort, fewer complications, and a high union rate.

Hak et al. [5] studied the comparison of single-plane and bi-plane external fixators for arthrodesis. They studied 17 and 19 cases, respectively, in each group. They demonstrated a decreasing fusion rate with an increasing number of previous surgeries. Single and bi-plane fixators had near similar fusion rates (58% and 65%, respectively). They commented that arthrodesis is difficult to achieve in patients with significant bone loss. Brodersen et al. [6] showed arthrodesis by an external fixator in 45 failed TKR cases. They proved that the technique of arthrodesis does not seem to influence the final result. The device was kept for an average of 10 weeks followed by cast immobilization until arthrodesis in their study.

MacDonald et al. [7] showed a variety of techniques for arthrodesis. Benhenneda et al. [8] studied knee fusion using a compression clamp and a single-plane external fixator for infection. They retrospectively studied 30 patients with infected TKR and chronic sepsis as major indications. The mean follow-up in the study was 42.5 ± 23.6 months. They achieved fusion in 83% of cases with an average of 7.5 months. There were 14 males and 16 females in their study. Pin-track infection was the major complication reported in the study.

Bae et al. [9] demonstrated knee arthrodesis in nine patients (six having tuberculosis and three having infected TKR). The average age was 54 years with an average follow-up of 16.5 months. The fixator was kept for an average period of 4.4 months. They achieved arthrodesis in all nine cases.

Some studies have demonstrated knee arthrodesis by other methods or a combination of various methods such as dual plate construct [10], mono-rail external fixator [11], combination with crossed cannulated screws [12], and circular external fixator [13]. Lohith et al. [14] showed fusion in 5°-7°valgus and 5°-15° knee joint flexion. They concluded that compression arthrodesis is a successful technique, but new techniques such as dual plating, short-locked intramedullary nails, and arthroscopy-assisted fusion can provide more successful fusion. Eralp et al. [15] proved the importance of intraoperative imaging for Schanz screw placement and bone cuts to avoid malalignment.

Our study matched with the others in the form of the main indication being infections, a male preponderance, position of fusion, and method of arthrodesis. The time for fixator removal was higher in our series compared to others. The cost of two pins and two Charnley’s clamps is much less in comparison to other implants. The prospective nature of the study, variety of cases, and mid-term follow-up are the strengths of our study. A single-center study and fewer patients can be considered study limitations. A multicenter study with a large number of patients can provide a better assessment of the study outcomes.

## Conclusions

Knee arthrodesis is a method of choice for infective conditions (septic or tuberculous) involving the knee joint, failed TKA, advanced stage of rheumatoid arthritis, failed fixation surgery around the knee joint, and neuropathic joint for which other surgical options to preserve joint movements are not viable. Preoperative counseling regarding the duration of treatment and shortening are crucial for better outcomes. There are various methods for arthrodesis, including internal fixation, external fixation (uniplanar/bi-planar/circular), or a combination of both. The fusion by Charnley’s external clamps is easy, cost-effective, and comfortable for the patients. The Knee Society Score and the Functional Knee Score significantly improved after the surgery. The complications were relatively less and the success rate of fusion was higher with this treatment method. Hence, the take-home message is to use Charnley’s external fixation device (compression arthrodesis) in select cases to achieve better clinical and functional outcomes.
